# Testosterone Levels in Adolescents and Young Men with Type 1 Diabetes and Their Association with Diabetic Nephropathy

**DOI:** 10.3390/biology10070615

**Published:** 2021-07-02

**Authors:** Jeongwoo Kang, Han Saem Choi, Young Ha Choi, Jun Suk Oh, Kyungchul Song, Junghwan Suh, Ahreum Kwon, Ho-Seong Kim, Hyun Wook Chae

**Affiliations:** Department of Pediatrics, Yonsei University College of Medicine, Seoul 06273, Korea; jeong.w.kang@yuhs.ac (J.K.); hansaem6890@yuhs.ac (H.S.C.); youngha@yuhs.ac (Y.H.C.); joojang87@yuhs.ac (J.S.O.); endosong@yuhs.ac (K.S.); suh30507@yuhs.ac (J.S.); armea@yuhs.ac (A.K.); kimho@yuhs.ac (H.-S.K.)

**Keywords:** adolescents, diabetic complications, diabetes mellitus type 1, diabetic nephropathy, testosterone

## Abstract

**Simple Summary:**

Type 2 diabetes(T2D) has been known to be related with obesity, insulin-resistance, impaired glucose control. Low testosterone levels and hypogonadism are also known as clinical characteristics of T2D patients. On the contrary, type 1 diabetes(T1D) happens when insulin is insufficient rather than insulin-resistance. Relationship between T1D and testosterone has not been established enough. In the study, patients with T1D showed higher testosterone levels than the general population. We could also find that higher testosterone levels have positive relationship with nephropathy, one of complications in diabetic patients. Therefore, periodic check-up for testostrone levels may be helpful for preventing nephropathy in T1D.

**Abstract:**

The association between serum testosterone levels and type 1 diabetes (T1D), especially in adolescents and young adults, has not been fully investigated. We aimed to compare testosterone levels between adolescents/young men with T1D and controls and to determine the factors affecting testosterone levels. We enrolled 47 men with T1D and 32 controls aged 15–29 years. We evaluated anthropometric measurements, lipid profiles, diabetic complications, and levels of serum luteinizing hormone, follicle-stimulating hormone, hemoglobin A1c, 24-h urine albumin, insulin autoantibody, and total serum testosterone. We assessed the correlation between serum testosterone levels and clinical characteristics. Total testosterone levels were higher in T1D patients than in controls (694.6 ± 182.2 vs. 554.1 ± 147.3 ng/dL, *p* = 0.001), and 24-h urine albumin level positively correlated with total testosterone levels (correlation coefficient 0.415, *p* = 0.004). T1D patients with nephropathy showed higher total testosterone levels than those without nephropathy (778.4 ± 198.9 vs. 655.4 ± 162.5 ng/dL, *p* = 0.029). However, diabetic nephropathy and testosterone levels were not significantly associated after adjusting for confounders (β ± SE 77.5 ± 55.2, *p* = 0.169). Further longitudinal studies are imperative to confirm a causal relationship between testosterone levels and T1D.

## 1. Introduction

It is widely accepted that men with type 2 diabetes (T2D) tend to have lower testosterone levels than the general population [1] Reduced testosterone levels are associated with reduced insulin sensitivity, i.e., insulin resistance in men with T2D [2,3] Previous studies have suggested that low testosterone levels are related to insulin resistance and elevated leptin levels in patients with T2D, especially in those with obesity [4,5].

Several studies have suggested that T2D is a risk factor for hypogonadism [6,7] The clinical characteristics of patients with T2D, such as obesity, dyslipidemia, impaired glucose regulation, and insulin resistance, were observed in men with hypogonadism. Hypogonadism in patients with T2D and metabolic syndrome has been associated with low serum testosterone levels [8,9] Recently, low testosterone levels have also been recognized as a predictor of cardiovascular disease, although the specific underlying mechanisms have not been entirely elucidated [10,11].

Although androgen insufficiency has been studied widely in patients with T2D, its association with type 1 diabetes (T1D) has not been clearly established. Danielson et al. [12] reported that men with T1D have similar total testosterone levels, high sex hormone-binding globulin levels, and slightly low free testosterone levels than those seen in controls. Contrastingly, Rohrer et al. [13] recently revealed a delay in the mean age at the onset of genital development and pubarche in boys with T1D. Likewise, the association between serum testosterone levels and T1D, especially in adolescents and young adults, has not been fully investigated.

Thus, this study aimed to compare the total testosterone levels in adolescents and young men with T1D with those in adolescents and young men without T1D. We also aimed to determine the factors affecting testosterone levels and examine their relationships. We hypothesized that young men with T1D have different testosterone concentrations than those with T2D and that testosterone concentration is related to clinical factors, such as body mass index (BMI), lipid profile, and diabetic complications, including nephropathy.

## 2. Materials and Methods

### 2.1. Participants

**Figure 1:**
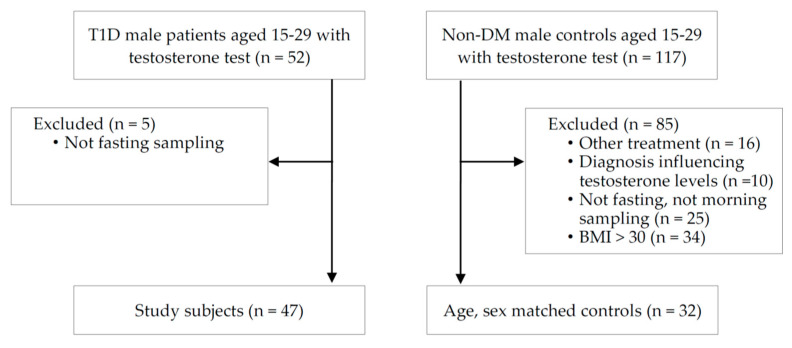
Summary of patient enrollment process and study design. T1D, type 1 diabetes; BMI, body mass index; DM, diabetes mellitus.

A total of 79 adolescents and young men among 169 eligible participants aged 15–29 years were enrolled. The calculated sample size was 42 based on the following factors: effect size, 0.5 (medium); α error probability, 0.05; power (1−β error probability), 0.70; and allocation ratio, N2/N1 (using the G Power software, version 3.1.9.4 (Heinrich-Heine-Universitat, Dusseldorf, Germany)). The retrospective nature of this study imposed a limitation on our attempt to enroll as many participants as possible. Overall, the study group included 47 patients diagnosed with T1D for >10 years, and the control group included 32 healthy participants without diabetes. The exclusion criteria for the control group were as follows: (1) history of sex hormone treatment; (2) diagnosis of any condition suspected of influencing testosterone levels, such as hypogonadism and delayed puberty; and (3) obesity, defined as a BMI exceeding two standard deviations (SD) from the normal BMI. The process of participant enrollment and study designs are presented in Figure 1.

### 2.2. Laboratory Measurements

Total testosterone levels were measured using the radioimmunoassay method (Diagnostic Products Corporation, Los Angeles, CA, USA), from blood samples that were collected in the morning after an overnight fast. The established reference range of total testosterone level in young Korean men is 300–600 ng/dL [14,15] Follicle-stimulating hormone (FSH) levels (DIAsource Immunoassays S.A., Ottignies-Louvain-la-Neuve, Belgium), luteinizing hormone (LH) levels (DIAsource Immunoassays, Belgium), and lipid profiles, including cholesterol and triglyceride levels, were also measured with the intent of establishing their associations with testosterone levels and diabetes. Hemoglobin A1c (HbA1c) levels were measured at baseline and every 6 months. The average HbA1c level was calculated using measurements made from the time of diagnosis until study commencement.

Nephropathy was defined based on the American Diabetes Association 2020 guidelines [16] Nephropathy was assessed based on the consecutive analyses of albumin excretion rate (>30 mg/24 h) or albumin-to-creatinine ratio (ACR, >30 mg/g), measured using spot urine samples. The presence of anti-glutamic acid decarboxylase antibodies (DIAsource Immunoassays) was analyzed at diagnosis.

### 2.3. Statistical Analyses

Data were analyzed using the SAS program, version 9.4 (SAS Institute, Cary, NC, USA). All data are baseline measurements and expressed as means ± SD for normally distributed values. Differences in normally distributed variables were tested using the *t*-test and analysis of variance (ANOVA), whereas differences in non-normally distributed variables were tested using the Kruskal–Wallis test. Variables related to testosterone levels were analyzed using Pearson’s correlation analysis. To determine the association between nephropathy and testosterone levels, multiple linear and logistic regression models were applied. A *p*-value of <0.05 was considered statistically significant.

### 2.4. Ethics Statement

All medical records were reviewed and analyzed retrospectively. The study was approved by the institutional review board of Yonsei University Severance Hospital (IRB number 3-2019-0199). The requirement of patient consent was waived because of the retrospective nature of the study.

## 3. Results

The baseline characteristics, including age, physical measurements, hormone levels, and lipid profiles, stratified by the study group and control group are presented in Table 1. Age, height, weight, BMI, and the levels of FSH, LH, blood urea nitrogen (BUN), and serum creatinine were not significantly different between the two groups. Total testosterone levels in adolescents and young men with T1D were higher than those in the control group (694.6 ± 182.2 ng/dL vs. 554.1 ± 147.3 ng/dL, *p* = 0.001).

**Figure 2:**
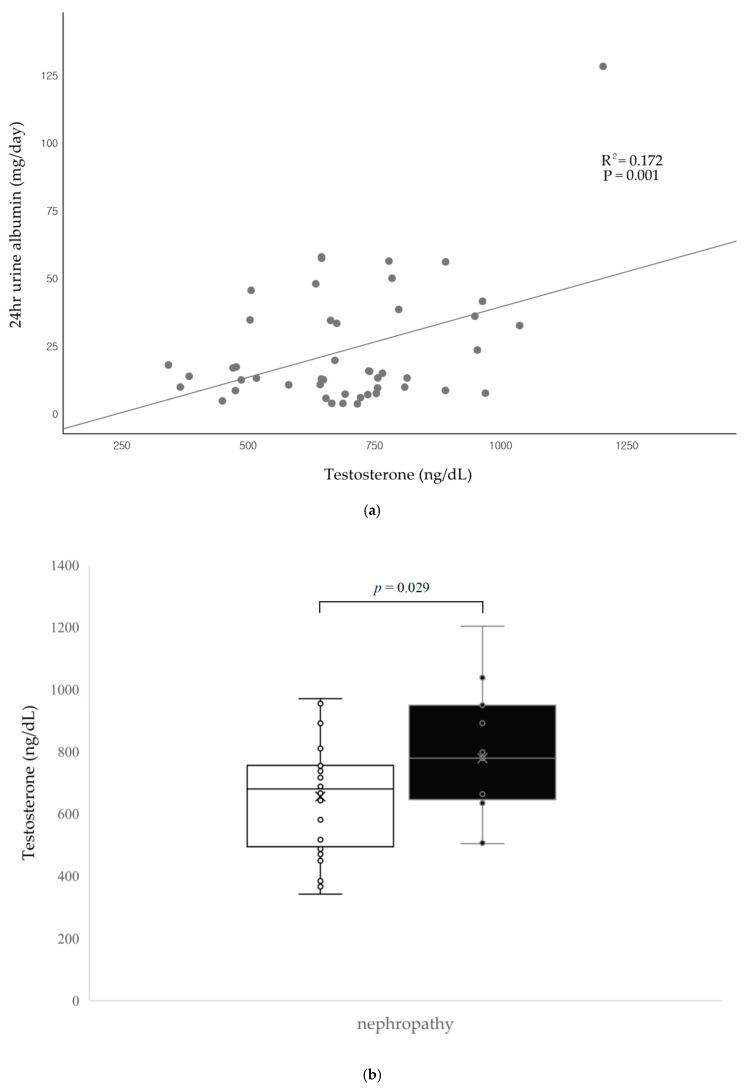
(**a**) The scatter plot and trend curve between total testosterone and 24 h urine albumin levels. (**b**) Testosterone levels of the study group according to nephropathy status. White, nephropathy; black, no nephropathy.

Pearson’s correlation analysis was performed to investigate the association of clinical parameters with testosterone levels and nephropathy (Table 2). Age, BMI, insulin dose, and the levels of total cholesterol, low-density lipoprotein (LDL) cholesterol, triglyceride, BUN, serum creatinine, HbA1c, and C-peptide did not significantly correlate with total testosterone levels. However, age, disease duration, and total testosterone levels significantly correlated with nephropathy. Spot urine ACR showed a significant correlation with total testosterone levels (correlation coefficient 0.402, *p =* 0.005). The 24 h urine albumin levels also significantly correlated with total testosterone levels (correlation coefficient 0.415, *p =* 0.004). A scatter plot and trend curve in Figure 2a explains this relationship.

**Figure 3:**
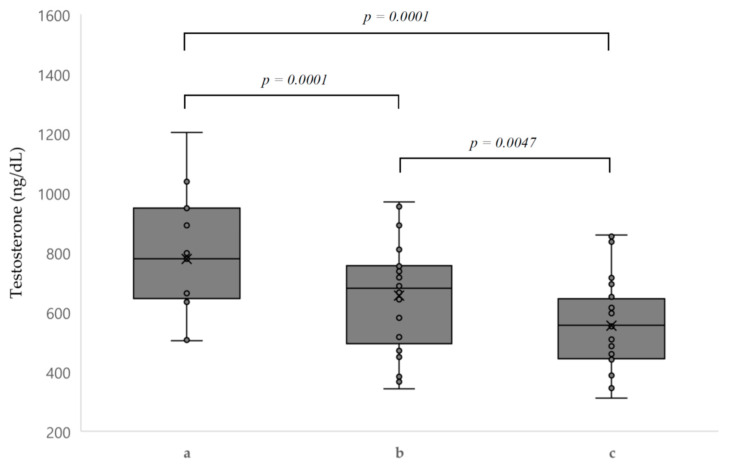
Boxplot of testosterone levels according to baseline characteristics. (a) Type 1 diabetes with nephropathy; (b) type 1 diabetes without nephropathy; and (c) control group.

We compared testosterone levels in patients with T1D using nephropathy status (Figure 2b). Patients with nephropathy showed higher testosterone levels than those without nephropathy (778.4 ± 198.9 ng/dL vs. 655.4 ± 162.5 ng/dL, *p* = 0.029). When comparing T1D patients with nephropathy, T1D patients without nephropathy, and the control group using ANOVA, it was found that total testosterone levels were the highest in patients with nephropathy and the lowest in controls (*p* = 0.001) (Figure 3).

Multiple linear regression analysis was performed to determine the relationship between total testosterone levels and nephropathy after adjusting for confounding factors. Nephropathy in patients with T1D did not show a significant relationship with testosterone levels after adjustment for age, BMI, LDL cholesterol levels, and HbA1c levels (*p* = 0.169) (Table 3).

## 4. Discussion

Although low testosterone levels have been associated with T2D as reported in previous studies, our findings were different in T1D patients. In our study, testosterone levels were not lower in the T1D group than in the control group. Among all factors, 24-h urine albumin levels and spot urine ACR were positively associated with testosterone levels. However, diabetic nephropathy, which was closely related to albuminuria, was not significantly associated with testosterone levels after adjusting for confounders.

Insulin resistance and elevated leptin levels in patients with T2D are known to be related to low testosterone levels [1,2] It has been proposed that insulin resistance causes hypogonadism through its relationship with leptin, an adipocyte-derived hormone and a recognized modulator of the hypothalamic–pituitary–gonadal axis [3,17] Hyperglycemia, caused by insulin resistance, induces an increase in leptin levels to reduce blood glucose levels [18,19] Persistently elevated leptin levels reported in patients with T2D, especially in obese men, may be responsible for the suppression of gonadal function [20,21] Testosterone levels in T1D patients are different from those in T2D patients because T1D is related to insulin deficiency rather than insulin resistance. Another mechanism not related to insulin or leptin may exist with respect to testosterone levels in T1D patients.

A few studies have investigated testosterone levels in patients with T1D. Hylmarova et al. [22] showed no significant changes in total testosterone levels in men with T1D; however, total testosterone levels were mainly associated with BMI. Holt et al. [23] reported that men with T1D do not appear to have a relatively high prevalence of androgen deficiency; hence, their testosterone levels are comparable to those of the general population. Ximena et al. [24] suggested that boys with T1D had younger age at onset of puberty and at the final stages of puberty than boys in the control group. This implies that higher testosterone levels may be present during puberty in boys with T1D. Condorelli et al. [25] also found that total testosterone levels were higher in patients with T1D than in those with T2D and control participants (610 ± 110 ng/dL in patients with T1D, 410 ± 50 ng/dL in patients with T2D, and 510 ± 10 ng/dL in control participants). Similar to the findings of the aforementioned studies, in our study, testosterone levels were not lower in patients with T1D than in control participants.

The finding that patients with T1D have high serum testosterone levels is not a general concept, based on previous studies. One of the hypotheses is that a lower BMI in T1D patients could result in higher total testosterone levels. T1D patients are underweight and have a lower BMI than the reference population at diagnosis [26]. Contrastingly, T2D patients have clinical characteristics such as obesity, higher BMI, and hypogonadism. Obesity with T2D leads to a greater chance of developing hypogonadotropic hypogonadism [27]. Therefore, an inverse association between BMI and total testosterone may induce higher testosterone levels in T1D patients. A higher BMI in the control group also supports the theory. The second hypothesis is that iatrogenic hyperinsulinemia may increase testosterone levels in T1D patients [28]. This may happen because T1D patients need a greater amount of insulin for glucose control than healthy controls with normal beta cells. Exogenous hyperinsulinemia in T1D patients may stimulate testosterone production and induce high serum testosterone levels. The fundamental mechanism underlying high testosterone levels in T1D patients has not yet been studied sufficiently. Thus, further studies are needed to elucidate the possible mechanisms involved.

We also investigated the potential factors influencing testosterone levels in patients with T1D. Wagner et al. [29] found that obesity and metabolic diseases during childhood and adolescence negatively influenced reproductive functions and, consequently, testosterone levels. Hart et al. [30] reported that the features of metabolic syndrome in late adolescence were associated with reductions in serum testosterone levels in the presence of insulin resistance. Chillarón et al. [31] revealed that low testosterone levels were related to age, waist circumference, and insulin requirements. In contrast, Condorelli et al. [25] reported that metabolic control was not related to testosterone levels in boys with T1D. In our study, BMI, HbA1c levels, and metabolic markers such as triglyceride levels showed a tendency toward negative correlation with total testosterone levels. The result supports the previously mentioned hypothesis that obesity and a higher BMI can be factors that decrease testosterone levels.

The association between testosterone levels and one of the complications of T1D, nephropathy, was investigated. It was noted that 24-h urine albumin levels and spot urine ACR had positive associations with total testosterone levels. Furthermore, patients with nephropathy showed significantly higher testosterone levels than those without nephropathy. Williamson et al. [32] also suggested that testosterone may contribute to an increase in the presentation of diabetic nephropathy during puberty. Amin et al. [33] reported that hyperandrogenism, including elevated testosterone levels and poor glycemic control, accompanies the development of nephropathy during puberty in T1D patients. Schultz et al. [34] suggested that elevated testosterone levels could be the underlying cause of nephropathy since the increase in androgen levels accelerates the development of renal disease during puberty. Doublier et al. [35] developed a mouse model with nephropathy, which is associated with high testosterone levels, and demonstrated that testosterone could induce podocyte apoptosis via androgen receptor activation. These findings explain the possible association between diabetic nephropathy and high testosterone levels. Nevertheless, serum BUN and creatinine levels, other indicators of nephropathy, did not correlate with total testosterone levels and nephropathy. This may be because most participants have microalbuminuria rather than macroalbuminuria, and its mild severity is not enough to influence creatinine levels.

Regulation of total testosterone levels could be associated with a reduction in nephropathy risk. For instance, there was a relevant study reporting that statins could lower testosterone levels and slow down the progression of CKD. Statins showed a positive effect on nephropathy by lowering oxidative stress and inflammation and stabilizing atherosclerotic plaques. More detailed pathophysiology, the association between statins and nephropathy, and the therapeutic use of statins to prevent nephropathy in T1D patients could be investigated in further studies [36].

Previous studies were subject to several limitations, such as the inclusion of participants from specific age groups, lack of control groups, and an average disease duration that was inadequate to investigate the relationship between total testosterone levels and diabetic complications. Some studies on adolescents limited their focus to puberty and sexual development. The strength of our study was that we included age-matched controls and studied the relationship between testosterone levels and diabetic complications in patients with T1D who were followed up at our center for >10 years.

The limitations of our study included its retrospective nature, small sample size, and single-center setting. We attempted to investigate the changes in total testosterone levels during the disease course of T1D; however, we could not collect appropriate data because of the retrospective nature of our study. Sex hormone-binding globulin, a protein that may cause variations in total testosterone levels, was not considered in our study. Further studies on the relationship of this protein with free testosterone levels may strengthen the validity of our findings. Another limitation was the possibility of unidentified, underlying conditions in participants from the control group that could have influenced testosterone levels. To mitigate this limitation, we carefully reviewed the entire medical records of all control participants and excluded those with any suspicious problems.

## 5. Conclusions

In summary, our study demonstrated that testosterone levels were not lower in adolescents and young men with T1D than in healthy controls. Total testosterone levels showed a significant relationship with 24-h urine albumin level and spot urine ACR in patients with T1D. This relationship suggests that T1D patients with diabetic nephropathy and those with microalbuminuria have higher testosterone levels than those without nephropathy. However, after adjusting for confounders, total testosterone levels were not significantly associated with nephropathy. Further longitudinal studies are warranted to confirm the presence of a causal relationship between testosterone levels and T1D.

## Figures and Tables

**Table 1:** Comparison of baseline characteristics between the type 1 diabetes and control groups.

	T1D (N = 47)	Control Group (N = 32)	*p*-Value
Age (year)	20.2 ± 4.4	19.6 ± 2.9	0.486
Disease duration (year)	11.3 ± 4.3	-	-
Height (cm)	172.7 ± 5.3	170.4 ± 8.7	0.288
Weight (kg)	63.9 ± 8.8	65.4 ± 10.2	0.563
BMI (kg/m^2^)	21.3 ± 2.4	22.9 ± 3.2	0.064
Testosterone (ng/dL)	694.6 ± 182.2	554.1 ± 147.4	0.001
FSH (IU/L)	3.6 ± 1.4	4.0 ± 2.1	0.427
LH (IU/L)	3.9 ± 1.3	3.8 ± 1.3	0.723
Total cholesterol (mg/dL)	147.7 ± 26.6	168.4 ± 32.5	0.003
LDL cholesterol (mg/dL)	91.6 ± 25.7	104.0 ± 31.2	0.064
Triglycerides (mg/dL)	81.8 ± 56.8	91.6 ± 40.7	0.422
Glucose (mg/dL)	156.8 ± 38.1	88.8 ± 12.5	0.001
BUN (mg/dL)	12.7 ± 3.1	11.2 ± 3.3	0.053
Serum creatinine (mg/dL)	0.9 ± 0.1	0.9 ± 0.1	0.607
HbA1c at diagnosis (%)	12.0 ± 4.4	-	-
Average HbA1c (%)	7.9 ± 1.2	-	-
C-peptide (ng/mL)	0.5 ± 0.2	-	-
24-h urine albumin (mg/day)	23.2 ± 22.8	-	-
Spot urine ACR (mg/g)	18.6 ± 39.6	-	-

T1D, type 1 diabetes; BMI, body mass index; FSH, follicle-stimulating hormone; LH, luteinizing hormone; LDL, low-density lipoprotein; BUN, blood urea nitrogen; HbA1c, hemoglobin A1c; ACR, albumin-to-creatinine ratio.

**Table 2:** Pearson’s correlation analysis for testosterone levels, nephropathy, and clinical variables in the study population.

Variable	Testosterone		Nephropathy	
	Correlation Coefficient	*p*-Value	Correlation Coefficient	*p*-Value
Age (year)	0.208	0.162	0.309	0.035
Disease duration (year)	0.118	0.429	0.353	0.015
BMI (kg/m^2^)	−0.184	0.238	0.225	0.146
Total cholesterol (mg/dL)	0.105	0.501	0.187	0.229
LDL cholesterol (mg/dL)	0.203	0.191	0.174	0.264
Triglycerides (mg/dL)	−0.069	0.658	0.046	0.769
BUN (mg/dL)	0.055	0.713	0.026	0.865
Serum creatinine (mg/dL)	−0.048	0.755	−0.106	0.494
HbA1c (%)	−0.040	0.787	−0.064	0.669
HbA1c at diagnosis (%)	0.075	0.623	0.048	0.755
Average HbA1c (%)	−0.121	0.416	−0.073	0.624
C-peptide (ng/mL)	0.130	0.385	−0.042	0.780
Insulin dose (unit/kg/day)	−0.229	0.121	−0.213	0.151
24-h urine albumin (mg/day)	0.415	0.004	0.802	0.001
Spot urine ACR (mg/g)	0.402	0.005	0.342	0.018
Testosterone (ng/dL)	-	-	0.318	0.029

BMI, body mass index; LDL, low-density lipoprotein; BUN, blood urea nitrogen; HbA1c, hemoglobin A1c.

**Table 3:** Multiple linear regression analysis of testosterone levels according to the presence of nephropathy.

Parameter	Model 1		Model 2		Model 3		Model 4	
	β ± SE	*p*-Value	β ± SE	*p*-Value	β ± SE	*p*-Value	β ± SE	*p*-Value
Age	-	-	6.4 ± 6.6	0.340	15.0 ± 6.6	0.030	-	-
Nephropathy	94.9 ± 55.2	0.093	74.9 ± 58.9	0.208	77.5 ± 55.2	0.169	115.1 ± 55.7	0.046
BMI (kg/m^2^)	-	-	-	-	−37.7 ± 11.9	0.003	−31.2 ± 12.2	0.015
LDL cholesterol (mg/dL)	1.1 ± 1.0	0.289	0.9 ± 1.0	0.143	0.9 ± 1.0	0.147	1.4 ± 1.0	0.020
Average HbA1c (%)	−7.7 ± 22.0	0.727	−9.4 ± 22.1	0.674	−48.5 ± 24.7	0.058	−40.1 ± 25.8	0.129

BMI, body mass index; LDL, low-density lipoprotein; HbA1c, hemoglobin A1c. Model 1, adjusted for LDL cholesterol and HbA1c levels; Model 2, adjusted for age, LDL cholesterol levels, and HbA1c levels; Model 3, adjusted for age, BMI, LDL cholesterol levels, and HbA1c levels; Model 4, adjusted for BMI, LDL cholesterol levels, and HbA1c levels.

## Data Availability

No new data were created or analyzed in this study. Data sharing is not applicable to this article.
